# Determination and Comparison of Seed Oil Triacylglycerol Composition of Various Soybeans (*Glycine max* (L.)) Using ^1^H-NMR Spectroscopy

**DOI:** 10.3390/molecules181114448

**Published:** 2013-11-21

**Authors:** Won Woo Kim, Ho Sik Rho, Yong Deog Hong, Myung Hun Yeom, Song Seok Shin, Jun Gon Yi, Min-Seuk Lee, Hye Yoon Park, Dong Ha Cho

**Affiliations:** 1 College of Biomedical Science, Kangwon National University, Chuncheon 200-701, Korea; E-Mail: jidoone@naver.com; 2 R & D Center, AmorePacific Corporation, Yongin 446-729, Korea; E-Mails: hydhong@amorepacific.com (Y.D.H.); mhyeom@amorepacific.com (M.H.Y.); ssshin@amorepacific.com (S.S.S.); 3 AmorePacific Group, Seoul 100-230, Korea; E-Mail: jgyi@amorepacific.com; 4 Sulloccha Research Center, Jangwon. Co., Ltd., Jeju 699-924, Korea; E-Mail: leems@jwgreent.co.kr; 5 National Institute of Biological Resources, Incheon 404-708, Korea; E-Mail: rejoice0777@gmail.com

**Keywords:** *Glycine max* (L.), soybean, seed oil, triacylglycerol, ^1^H-NMR

## Abstract

Seed oil triacylglycerol (TAG) composition of 32 soybean varieties were determined and compared using ^1^H-NMR. The contents of linolenic (Ln), linoleic (L), and oleic (O) ranged from 10.7% to 19.3%, 37.4%–50.1%, and 15.7%–34.1%, respectively. As is evident, linoleic acid was the major fatty acid of soybean oil. Compositional differences among the varieties were observed. Natural oils containing unsaturated groups have been regarded as important nutrient and cosmetic ingredients because of their various biological activities. The TAG profiles of the soy bean oils could be useful for distinguishing the origin of seeds and controlling the quality of soybean oils. To the best of our knowledge, this is the first study in which the TAG composition of various soybean oils has been analyzed using the ^1^H-NMR method.

## 1. Introduction

Nowadays, soybean and its products are attracting more attention because of their beneficial effects on human health [1]. The most important organic compound of the soybean seed are proteins (approximately 40%) and oil (approximately 20%). Soybean oil is extensively used as cooking oil. The main component of natural oils, including soybean oil, is triacylglycerol (TAG), which contains unsaturated functional groups such as linolenic (Ln), linoleic (L), and oleic (O) acids [2]. The nutritional value and physiochemical properties of natural oil are linked to the composition of TAG. Recently, various biological activities of natural oils on the skin have been reported [3,4,5,6]. Their activities might be caused by the unsaturated groups. Soy bean oils have a great potential as active cosmetic ingredients because of the presence of a large quantity of unsaturated functional groups. Therefore, defining the TAG composition of oil is very important because it provides information about the quality of the oil. The contents and compositions of TAG may depend on the condition of cultivation and varieties [7,8]. There have been numerous studies on the determination of chemical compounds in soybean varieties [9,10,11]. However, the composition of oil from various soybean varieties had not been studied well [12]. Thus, the aim of this study was to determine and compare the seed oil TAG composition of soybean varieties. After analyzing their TAG profiles, soybean varieties that were suitable for use as cosmetic ingredients were selected. Several methods, including gas-liquid chromatography, are available for the quantitative determination of TAGs in oil [13,14,15]. However, these methods are labor-intensive and time-consuming because of the complex series of reaction steps. Recently, proton nuclear magnetic resonance (^1^H-NMR) has been used as an alternative analytical method to provide information about the TAG composition [16,17,18]. We also selected ^1^H-NMR as an analytical method because it allows rapid, simultaneous, noninvasive, and nondestructive analysis without chemical reactions. Thirty two varieties of soybean cultivated in Korea were chosen for this study.

## 2. Results and Discussion

The soybean seeds contained 0.9%–11.9% of oil (Table 1). The highest amount of oil was measured in the Shinhwakong variety (11.9%), while the lowest content was observed in the Yak-kong variety (0.9%).

**Figure 1:**
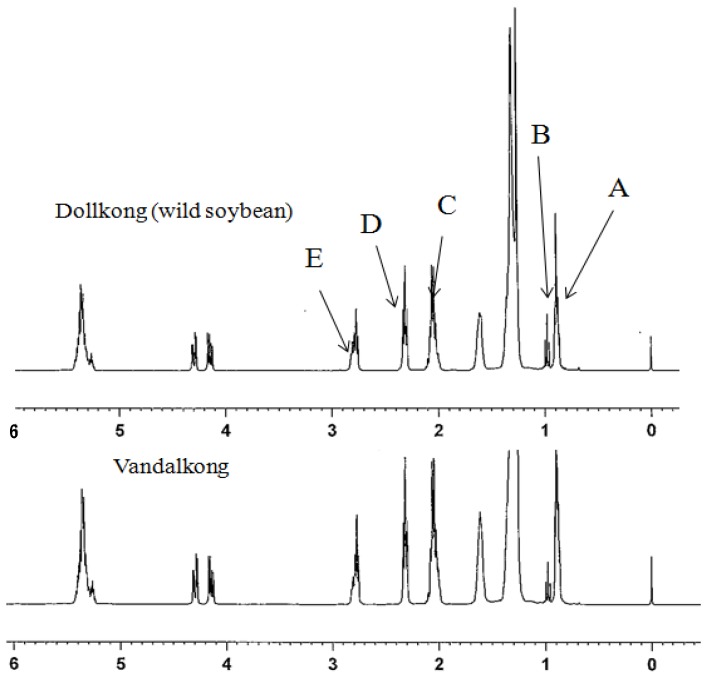
^1^H-NMR spectra of Dollkong (wild soybean) and Vandalkong oils.

We studied the 400 MHz spectra of the soybean oils. The ^1^H-NMR spectra of Dollkong (wild soybean) oil and Vandalkong oil are shown in Figure 1. The composition of Ln, L, O, and saturated (S) acyl groups can be determined using the following equations [17,18]:

Ln (%) = 100 [B/(A + B)],

L (%) = 100 [(E/D) – 2[B/(A + B)]],

O (%) = 100 [(C/2D) – (E/D) + [B/(A + B)]],

S (%) = 100 [1 − (C/2D)].

The various peaks are assigned as follows (Table 2): signal A is produced by the overlapping of the triplet signals of methyl group protons of S, O, and L acyl groups. Signal B is the triplet methyl protons of Ln acyl groups. Signal C is due to the α methylene protons in relation to a single double bond. Signal D is due to the methylene protons in the α position in relation to the carboxyl group. Signal E is due to the overlapping of the signals from the α methylene protons in relation to two double bonds. The area of signal B obtained from Dollkong (wild soybean) oil is bigger than that of Vandalkong oil (Figure 1).

**Table 1:** Seed color and oil yield of 32 soybean varieties.

Entry	Variety	Color	Oil yield ^a^
1	Jangwonkong	yellow	5.4
2	Anpyungkong	yellow	7.3
3	Daepungkong	yellow/wine	6.5
4	Daewonkong	yellow/wine	8.5
5	Pureunkong	green	2.7
6	Samnamkong	green	4.8
7	Dooyookong	yellow	5.3
8	Cheongjakong	black	3.9
9	Buseok	green	4.2
10	Dachaekong	yellow	3.3
11	Seonamkong	yellow	3.5
12	Yak-kong	black	0.9
13	Hwangsekjilgeumkong	yellow/green	3.5
14	Milyangkong	yellow	2.3
15	Danwonkong	yellow	6.2
16	Bongeui	yellow	2.5
17	Kwangdu	yellow	4.1
18	Seomoktae	yellow	4.0
19	Subaktae	black/green	3.4
20	Seoritae	black	4.1
21	Shinhwakong	yellow	11.9
22	Vandalkong	wine	7.9
23	Saeal	black/green	5.1
24	Geomjeong Bul Kong	light black	6.4
25	Bamsekyak-kong	wine	8.7
26	Bamkong	wine/white spot	9.5
27	Yongan	black/light yellow	6.4
28	Horangi kong	wine	6.2
29	Seonbikong	black/light green	8.5
30	Dollkong (wild soybean)	dark wine	2.3
31	Nabdegikong	wine/light green	7.2
32	Geomjeong-nabdegikong	black	3.8

^a^ The oil yield is expressed as % (w/w) on a dry weight basis.

**Table 2:** Assignment of signals of ^1^H-NMR spectra. Signal number agrees with those in Figure 1.

Signal	Chemical shift (ppm)	Functional group
A	0.83–0.93	-C**H**_3_ (saturated, oleic and linoleic acyl chains)
B	0.93–1.03	-C**H**_3_ (linolenic acyl chains)
C	1.94–2.14	-C**H**_2_-CH=CH- (acyl chains)
D	2.23–2.36	-OCO-C**H**_2_- (acyl chains)
E	2.70–2.84	=HC-C**H**_2_-CH= (acyl chains)

The acyl group compositions of 32 soybean oils obtained by ^1^H-NMR data are shown in Table 3. The content of Ln in the oils, containing three unsaturated groups, ranged from 10.7% to 19.3%. The highest content of Ln was measured in Dollkong (wild soybean) (19.3%). L was the major acyl group, with content ranging from 37.4% to 50.1%. The highest content of L was measured in the Vandalkong variety (50.1%), while the lowest content was observed in the Hwangsekjilgeumkong variety (37.4%). The mean content of L was 45.0%. The content of O ranged between 15.7% and 34.1%, and its mean content was 24.1%. The highest content of O was measured in the Hwangsekjilgeumkong variety (34.1%). The S content varied between 13.3% and 20.9%. The highest S content was measured in the Anpyungkong variety (20.9%), while the lowest was in the Samnamkong variety (13.3%).

**Table 3:** TAG composition of soybean oils using ^1^H-NMR analysis.

Entry	Variety	Linolenic	Linoleic	Oleic	Saturated
1	Jangwonkong	12.3%	44.2%	26.5%	17.0%
2	Anpyungkong	13.0%	48.0%	18.1%	20.9%
3	Daepungkong	13.8%	47.7%	23.2%	15.3%
4	Daewonkong	13.8%	45.6%	24.3%	16.3%
5	Pureunkong	12.3%	45.1%	23.4%	19.2%
6	Samnamkong	13.5%	40.8%	32.4%	13.3%
7	Dooyookong	12.3%	49.4%	22.5%	15.8%
8	Cheongjakong	12.3%	44.3%	22.5%	15.8%
9	Buseok	13.8%	41.4%	25.8%	19.0%
10	Dachaekong	13.0%	45.3%	24.7%	17.0%
11	Seonamkong	13.0%	43.0%	27.8%	16.2%
12	Yak-kong	13.0%	43.7%	28.1%	15.2%
13	Hwangsekjilgeumkong	12.2%	37.4%	34.1%	16.3%
14	Milyangkong	11.5%	38.2%	31.5%	18.8%
15	Danwonkong	10.7%	49.0%	26.2%	14.0%
16	Bongeui	13.0%	39.4%	31.5%	16.1%
17	Kwangdu	13.0%	45.9%	25.7%	15.4%
18	Seomoktae	12.3%	47.9%	21.0%	18.8%
19	Subaktae	13.0%	46.9%	23.8%	16.3%
20	Seoritae	13.0%	45.8%	24.6%	16.6%
21	Shinhwakong	13.0%	45.5%	25.2%	16.3%
22	Vandalkong	12.3%	50.1%	16.8%	20.8%
23	Saeal	15.2%	43.3%	21.8%	19.7%
24	Geomjeong Bul Kong	12.2%	43.3%	25.7%	18.8%
25	Bamsekyak-kong	12.2%	47.8%	22.1%	17.9%
26	Bamkong	15.2%	36.5%	29.8%	18.5%
27	Yongan	13.8%	47.4%	18.5%	20.3%
28	Horangi kong	13.0%	49.2%	20.8%	17.0%
29	Seonbikong	14.5%	45.9%	22.2%	17.4%
30	Dollkong (wild soybean)	19.3%	45.6%	15.7%	19.4%
31	Nabdegikong	15.9%	48.8%	18.5%	16.8%
32	Geomjeong-nabdegikong	15.2%	48.5%	18.3%	18.0%
	Mean	13.3%	45.0%	24.1%	17.3%

## 3. Experimental

### 3.1. Soybean Materials

The soybean varieties purchased and used for this experiment had been grown in Gangwon-do, Chuncheon, South Korea in 2012. Soybean seeds were soaked in water, then washed to remove any adhering flesh and finally oven dried at 40 °C for 24 h. The dried seeds were crushed for use.

### 3.2. Preparation of Oils

Soybean oils were prepared through a pressing method. Soybean oil was extracted using a breast pump (HD-333, Hyun-dae Green Industrial Co., Seoul, Korea). The oil was centrifuged (VS-5000N, Vision Scientific Co., Seoul, Korea) at 3,000 rpm for 10 min, and then supernatant were collected for analysis.

### 3.3. ^1^H-NMR Analysis

^1^H-NMR analyses were performed on Varian Mercury 400 (400 MHz for ^1^H) instrument. Each oil sample, weighing 0.2 g, was dissolved in CDCl_3_ (400 µL, Sigma-Aldrich, Saint Louis, Missouri, USA) with a small amount of TMS as internal standard and the resulting mixture was placed into a 5-mm diameter ultra-precision NMR sample tubes. The temperature of the sample in the probe was 30 °C. Chemical shifts were recorded in ppm, using the solvent proton signal as standard. The area of the signals was determined by using the equipment software.

## 4. Conclusions

Oil yield and TAG composition of 32 soybean varieties were studied. ^1^H-NMR was selected as the analytical method. Quantitative differences in the TAG profiles of various varieties were observed. The study of TAG profiles can be a convenient method of distinguishing the origin of seeds and controlling the quality of soybean oils. After analyzing the TAG profiles, three varieties (Dollkong, Vandalkong, and Hwangsekjilgeumkong) were chosen as potentially effective cosmetic ingredients. The highest content of Ln was measured in Dollkong (wild soybean) (19.3%). The highest content of L was measured in the Vandalkong variety (50.1%). The highest content of O was measured in the Hwangsekjilgeumkong variety (34.1%). Further studies on their biological activities on the skin are underway.
